# Donor-Derived Myeloid Heme Oxygenase-1 Controls the Development of Graft-Versus-Host Disease

**DOI:** 10.3389/fimmu.2020.579151

**Published:** 2021-01-18

**Authors:** Chloé Spilleboudt, Virginie De Wilde, Philippe Lewalle, Ludovic Cabanne, Mathieu Leclerc, Florence Beckerich, Dominique Bories, Silvia Cardoso, Miguel P. Soares, Benoît Vokaer, Jean-Michel Hougardy, Véronique Flamand, Judith Racapé, Marc Abramowicz, Sébastien Maury, Alain Le Moine

**Affiliations:** ^1^ Institute for Medical Immunology, Université Libre de Bruxelles, Gosselies, Belgium; ^2^ Erasme Hospital, Hematology Department, Université libre de Bruxelles, Brussels, Belgium; ^3^ Jules Bordet Institute, Hematology Department, Université libre de Bruxelles, Brussels, Belgium; ^4^ AP-HP, Hôpital Henri Mondor, Department of Hematology, Créteil, France; ^5^ University Paris Est Créteil (UPEC), Créteil, France; ^6^ Instituto Gulbenkian de Ciência, Oeiras, Portugal; ^7^ Erasme Hospital, Nephrology and Internal Medicine Department, Université libre de Bruxelles, Brussels, Belgium; ^8^ Centre de Recherche Épidémiologie, Biostatistique et Recherche clinique, École de Santé Publique, Université libre de Bruxelles, Brussels, Belgium; ^9^ Department of Genetic Medicine and Development, Faculty of Medicine, University of Geneva, Geneva, Switzerland

**Keywords:** Heme oxygenase-1, graft-versus-host disease, transplantation, hematopoeietic stem cell transplantation, polymorphism, myeloid-derived suppressor cells

## Abstract

Graft-versus-host disease (GVHD) remains a major clinical drawback of allogeneic hematopoietic stem cell transplantation (HSCT). Here, we investigated how the stress responsive heme catabolizing enzyme heme oxygenase-1 (HO-1, encoded by *HMOX1*) regulates GVHD in response to allogeneic hematopoietic stem cell transplantation in mice and humans. We found that deletion of the *Hmox1* allele, specifically in the myeloid compartment of mouse donor bone marrow, promotes the development of aggressive GVHD after allogeneic transplantation. The mechanism driving GVHD in mice transplanted with allogeneic bone marrow lacking HO-1 expression in the myeloid compartment involves enhanced T cell alloreactivity. The clinical relevance of these observations was validated in two independent cohorts of HSCT patients. Individuals transplanted with hematopoietic stem cells from donors carrying a long homozygous (GT)_n_ repeat polymorphism (L/L) in the *HMOX1* promoter, which is associated with lower HO-1 expression, were at higher risk of developing severe acute GVHD as compared to donors carrying a short (GT)_n_ repeat (S/L or S/S) polymorphism associated with higher HO-1 expression. In this study, we showed the unique importance of donor-derived myeloid HO-1 in the prevention of lethal experimental GVHD and we corroborated this observation by demonstrating the association between human *HMOX1* (GT)_n_ microsatellite polymorphisms and the incidence of severe acute GVHD in two independent HSCT patient cohorts. Donor-derived myeloid HO-1 constitutes a potential therapeutic target for HSCT patients and large-scale prospective studies in HSCT patients are necessary to validate the HO-1 L/L genotype as an independent risk factor for developing severe acute GVHD.

## Introduction

Acute graft-versus-host disease (GVHD) remains a major cause of mortality after allogeneic HSCT (1, 2). This pathologic process is counter-regulated by regulatory T cells (T_regs_) (3, 4) as well as by myeloid-derived suppressor cells (MDSCs) (5, 6). The latter are a heterogeneous population of immature myeloid cells harboring monocytic or polymorphonuclear markers (7–10), that suppress T cell responses *via* diverse and not fully understood mechanisms. In an experimental mouse model, MDSCs suppress acute GVHD *via* an IL-13- and arginase-1-dependent mechanism that regulates graft-versus-host alloreactivity (11). In other experimental settings, MDSCs have also been show to exert T cell suppression through indoleamine 2,3-dioxygenase (IDO) production or through nitric oxide synthase (NOS)-dependent mechanisms which are associated or not with the expression of heme oxygenase-1 (HO-1) (12–14). Of note, IDO and iNOS are heme proteins that are tightly regulated by mechanisms that control heme-iron metabolism in monocyte cells (15). Moreover, heme catabolism by HO-1 counter-regulates the rejection of transplanted cells, tissues, or organs in a variety of experimental conditions (16). In keeping with this notion, we have previously shown that MDSCs block T cell-mediated allograft rejection *via* a mechanism that is dependent on the expression of HO-1 (17). It has also been reported that non-specific pharmacologic HO-1 overexpression regulates T cell alloreactivity in a manner that prevents the onset of experimental GVHD (18–21). However, whether natural endogenous levels of HO-1 limit GVHD and its sources, and the contribution of donor versus recipient HO-1 remain critical open questions.

The human *HMOX1* promoter has a microsatellite polymorphism in which the number of (GT) repeats (GT)_n_ is inversely correlated with *HMOX1* transcriptional activity and, ultimately, HO-1 expression (22). This (GT)_n_ polymorphism has been associated with the clinical outcome of a variety of inflammatory diseases (23, 24). Specifically, longer (GT)_n_ repeats are associated with low levels of HO-1 expression (21) and correlate with higher disease susceptibility, while shorter (GT)_n_ repeats are associated with higher levels of HO-1 expression and correlate with lower disease susceptibility. This has been illustrated for cardiovascular disease (25–28), chronic kidney disease (29, 30), HIV-induced central nervous system neuroinflammation (31, 32), rheumatoid arthritis (33), chronic obstructive pulmonary disease (34), and organ transplantation (35, 36). In sharp contrast, however, shorter (GT)_n_ repeats are associated with higher levels of HO-1 expression and promote metaflammation associated with the onset of experimental metabolic disease (37).

Herein, we demonstrated the unique importance of donor-derived myeloid HO-1 in the prevention of lethal experimental GVHD and we corroborated this observation with the association between the human *HMOX1* (GT)_n_ microsatellite polymorphisms and the incidence of severe acute GVHD in two independent HSCT patient cohorts.

## Methods

### Mice

*Hmox1* ^−/−^ mice (38) were generated by *Hmox1* ^+/−^ mating and genotyped, as previously described (38, 39). C57BL/6 *LysM* ^Cre/wt^ *Hmox1^Δ/Δ^* mice were generated by mating of C57BL/6 *Hmox1* ^lox/lox^ mice (40) with C57BL/6 LysM^Cre/wt^ mice (kind gift from S.A. Nedospasov) (41). Mice received humane care in compliance with the Principles of Laboratory Animal Care formulated by the National Institutes of Health (Bethesda, MA), and protocols were approved by the local committee for animal welfare (agreement # LA2500519).

### Graft-Versus-Host Disease and *Ex Vivo* Assays

Recipients were lethally irradiated with 750cGy (BALB/c) or 980cGy (C57BL/6) from a 137Cs source, one day before bone marrow (BM) and whole spleen cell infusions (2.5 × 10^6^ BM and 2 × 10^6^ spleen C57BL/6 cells into BALB/c recipients and 5 × 10^6^ BM with 15 × 10^6^ spleen BALB/c cells into C57BL/6 recipients into the tail vein in 0.2 ml of RPMI solution). GVHD was monitored as previously described (42). For both CD8+ and CD4+ T cell depletion, 1 mg of a pair of monoclonal antibodies (mAbs) were injected i.p. at day 1 posttransplant (clone YTS 169 plus YTS 156 for CD8+ T cells and clones YTS191 plus YTA 3.1.2 for CD4+ T cells (43)) or YCATE mAb (control isotype), all kindly provided by Dr. S. Cobbold, Dunn School of Pathology, Oxford University, UK.

### Mixed Lymphocyte Reaction, Cytokine and HO-1 Quantification

For MLR, responder cells (2.5 × 10^6^ cells/ml) isolated from host spleens were stimulated with irradiated adequate splenocytes (2000 Rad; 2.5 × 10^6^ cells/ml), in 48-well, flat-bottom plates. IFN-γ, IL-17A, and IL-10 production was measured in the culture supernatants after 48 or 72 h using commercially available ELISA kits (Duoset, R&D Systems, Minneapolis, MN). The detection threshold was 10 pg/ml for IFN-γ, IL-17A, and IL-10. For HO-1 plasma quantification, the HO-1 ELISA kit (ADI-960-0F1, Enzo Life Sciences) was used following manufacturer instructions.

### RNA Extraction and Real-Time Reverse Transcription-PCR

Total RNA was extracted from liver and spleen using the MagNaPure LC RNA Isolation Kit III for tissue (Roche Diagnostics 03330591001) or from bone marrow-derived macrophages (BMDMs) with the MagNaPure LC RNA Isolation Kit-High Performance (Roche Diagnostics 03542394001). Reverse transcription and real-time PCR were performed using LightCycler-RNA Master Hybridization Probes (one-step procedure) on a LightCycler apparatus (Roche Diagnostics). The number of mRNA copies was evaluated using a standard curve for each gene of interest and was normalized to β-actin as a housekeeping gene. Primer and probe sequences are detailed in **Table S1**.

### Flow Cytometry and Other *Ex Vivo* Analyses

Commercially available fluorescent antibodies were used following manufacturer’s instructions. FITC-conjugated anti-mouse F4/80 (clone BM8), APC-conjugated anti-mouse H2kb (clone AF6-88.5.5.3), eFluor450-conjugated anti-mouse Ly-6G (clone RC57BL/6-8C5), eFluor450-conjugated anti-mouse H2kd (clone SF1-1.1.1), PerCP-Cy5.5-conjugated anti-mouse Ly-6C (clone HK1.4), and anti-mouse CD16/CD32 (Fc block, clone 93) were purchased from eBioscience. FITC-conjugated anti-mouse IA/IE (clone 2G9), PE-conjugated anti-mouse CD11c (cloneHL3), PE-conjugated anti-mouse CD49d (clone 9C10), PB-conjugated anti-mouse CD3 (clone 500A2), APC-conjugated anti-mouse CD8 (clone 53–6.7), and APC-conjugated anti-mouse CD11b (clone M1/70) were purchased from BD Biosciences.

HO-1 intracytoplasmic staining was performed using the Intracellular Fixation and Permeabilization Buffer Set (88–8824, eBioscience) and unconjugated anti-mouse HO-1 (clone HO-1-1, Abcam), followed by FITC-conjugated anti-mouse IgG1 or V450-conjugated anti-mouse IgG1(clone A85-1, BD Biosciences). The isotype clone NCG01 was used as a control antibody. Flow cytometry analysis was performed on a BD LSRFortessa cell analyzer using BD FACSDiva software (BD Biosciences).

Hemoglobin levels, blood leukocyte and platelet counts were performed using the automated system ADVIA 120 (Siemens, Munich, Germany).

Serum aspartate aminotransferase (AST) levels were measured by specific absorbance (Cobas 501, Roche Diagnostics).

### Generation of Bone Marrow-Derived Macrophages

1 × 10^6^ BM cells/ml isolated from femurs and tibias were cultured in DMEM medium supplemented with L-Glutamine, 4.5 g/L glucose (BE12-604F, Lonza), 10% heat-inactivated fetal calf serum (FCS), amino acids, sodium pyruvate, penicillin/streptomycin, β-mercaptoethanol, and 20% supernatant from an M-CSF-transfected L929 cell culture. Three days later, 5 ml of complete medium containing 60% of L929 cell supernatant was added. At day 7, BM-derived macrophages (BMDMs) were stimulated with 100 ng/ml of LPS (L2630, Sigma-Aldrich) for 24 h in fresh medium with 2% L929 supernatant.

### CFSE-Based Cytotoxicity Assay

After a wash with phosphate-buffered saline (PBS), target spleen cells were resuspended at 1 × 10^6^ cells/ml and labeled with 0.2 (BALB/C) or 2 µM (C57BL/6) of CFSE (Cell trace CFSE Cell Proliferation Kit) in pre-warmed PBS-BSA 0.1% for 10 min at 37°C. The reaction was stopped by the addition of an equal volume of cold PBS, followed by 5 min of incubation on ice. Then, 100 µl of CFSE-labeled cell solution (200 × 10^6^ cells/ml of BALB/C and C57BL/6 mice) was injected IV into mice with GVHD. Two hours later, spleen cells were analyzed by FACS.

### Study Population

Data were collected from two independent cohorts of 120 and 160 patients. The first cohort included 120 patients at the Henri Mondor hospital in Paris (France) who underwent HSCT between January 2007 and December 2012. The second cohort included 160 patients who underwent HSCT at the Jules Bordet Institute (Brussels, Belgium) between January 2001 and December 2011. In each cohort, all HSCT patients were retrospectively included during the fixed period without exclusion criteria. All grafts were from HLA-matched related donors (MRD) or HLA-matched unrelated donors (MUD). Patient and donor characteristics at the time of transplantation are shown in **Tables 1** and **2**. Patient characteristics in the two cohorts were compared using Pearson’s chi-square test for categorical data and Student’s t test for continuous variables distributed normally. Myeloablative regimens are detailed in the supplementary data. Early disease stage was defined as being either the first complete remission (CR) of acute leukemia or a chronic phase of chronic myeloid leukemia, whereas late disease stage included patients who did not achieve CR or were beyond the first CR.

**Table 1:** Patient characteristics.

Patient characteristics		
	Cohort 1 (n = 124)	Cohort 2 (n = 160)
Age (mean ± SD)	46.4 ± 13.5	40.8 ± 13.6
**Sex** n (%)		
Male	77 (62.7)	106 (66.25)
Female	47 (37.9)	54 (33.75)
**CMV** n (%)		
Positive	83 (66.9)	103 (64.4)
Negative Unknown	41 (33.1) 0	45 (28.1) 12 (7.5)
**Disease stage** n (%)		
CR1 or CML	64 (51.6)	72 (45.0)
>CR1	58 (46.7)	88 (55.0)
Not applicable	2 (1.6)	0
**Conditioning** n (%)		
MAC	44 (35.5)	92 (57.5)
RIC	80 (64.5)	68 (42.5)
**Graft** n (%)		
MRD	60 (48.4)	86 (53.8)
MUD	64 (51.6)	39 (24.4)
URD	0	35 (21.9)
**ATG** n (%)		
No	93 (75.0)	38 (23.8)
Yes	31 (25.0)	122 (76.2)
**Disease** n (%)		
AML/MDS/MF/ALL	85 (68.6)	98 (61.3)
Lymphoma	16 (12.9)	21 (13.1)
Multiple Myeloma	11 (8.9)	12 (7.5)
CML/CLL	8 (6.5)	20 (12.5)
Aplasia	2 (1.6)	5 (3.1)
Choriocarcinoma	0	1 (0.5)
Hemoglobinopathy	2 (1.6)	3 (2.0)

CMV indicates cytomegalovirus; CR1, first complete remission; CML, chronic myeloid leukemia; MAC, myeloablative conditioning; RIC, reduced intensity conditioning; MRD, matched related donor; MUD, matched unrelated donor; URD, unmatched related donor; ATG anti-thymocyte globulin; AML, acute myeloid leukemia; ALL, acute lymphoid leukemia; MDS, myelodysplastic syndrome; MPS, myeloproliferative syndrome; CLL, chronic lymphoid leukemia.

**Table 2:** HSC donor characteristics.

	Cohort 1 (n = 124)	Cohort 2 (n = 160)
Age (mean ± SD)	41 ± 14.0	40.1 ± 14.4
**Sex mismatch** (%)		
Female/Male	29 (23.4)	32 (21.8)
Others	95 (76.6)	115 (78.2)
**CMV** n (%)		
Positive	67 (54.0)	85 (53.1)
Negative Unknown	57 (46.0) 0	55 (33.4) 20 (12.5)

Acute GVHD was defined according to the National Institutes of Health consensus criteria based upon the timing of presentation, the typical clinical features, and the absence of diagnostic or distinctive features of chronic GVHD (44) and was graded according to the Glucksberg criteria (45). DNA samples from healthy volunteers were used for the control group.

### DNA Sample Collection

DNA samples were obtained from residual material. In controls, DNA samples were prepared from peripheral blood stem cells (PBSCs) using standard procedures and commercially available kits. Genetic analyses were retrospectively performed with the approval of the Ethics Committees of Erasme Hospital and the Jules Bordet Institute (numbers P2011/255 and 2012/193, respectively). The Henri Mondor University Hospital (Créteil, France) has been accredited according to the international JACIE program since 2005. All patients and donors signed informed consent for (1) registration into the Promise database, and (2) cryopreservation of their biological material for research purposes.

### HO-1 (GT)_n_ Polymorphisms and Genotypes

The 5′-flanking region containing a poly (GT)*_n_* repeat of the HO-1 gene was amplified by PCR. A FAM-labelled sense primer, 5′-AGA-GCC-TGC-AGC-TTC-TCA-GA-3′, and an antisense primer, 5′-ACA AAG-TCT-GGC-CAT-AGG-ACC-3′, were used to amplify 50 ng of genomic DNA. Primers were purchased from Eurogentec. PCR was performed over 20 touchdown cycles of denaturation at 94°C for 20 s, annealing at 62°C to 52°C (temperature decreasing by 0.5°C at each cycle) and extension at 72°C for 30 s. To determine fragment lengths, the PCR product was loaded onto a POP-6 matrix (Applied BioSystems), and calculations of fragment lengths were laser based through an automated fragment analyzer (model 3130XL, Applied BioSystems) and comparisons with sequenced alleles of different lengths (GS-500 ROX Applied BioSystems).

In relation to functional data on GT repeats described by others (22, 27), alleles were classified into two groups: short (S) alleles with less than 30 GT repeats, and long (L) alleles with more than 30 GT repeats. This classification entails three possible genotypes within the donor and host populations: S/S, S/L, and L/L.

### Statistical Analyses

To compare categorical data for allelic distribution, genotype distribution, Hardy-Weinberg equilibrium, and GVHD frequency, either Fisher’s exact test or Pearson’s chi-square test was used, as appropriate. The association between GVHD and risk factors was determined by univariate and multivariate analyses. When estimating cumulative incidence of GVHD, we took into account death without GVHD as a competing risk and used the Fine and Gray model to estimate its subdistribution hazard ratio (SHR). Since graft rejection was an exclusion criterion for the study, it was not considered as a competing event for GVHD occurrence. Multivariate analysis included variables significantly associated with GVHD severity, if shown by univariate analysis. Due to the number of cases and the collinearity between the donor and the recipient genotypes, only three variables were included in our model: graft, radiotherapy, and donor genotype. The adjusted SHRs (aSHRs) are reported. All statistical analyses were performed using STATA 12.0 software. A p*-*value <0.05 was considered statistically significant. For mouse experiments, differences between groups were estimated using the two-tailed Mann-Whitney nonparametric test. Mouse survival curves were compared using the log-rank test. A p-value <0.05 was considered statistically significant.

## Results

### Donor Myeloid HO-1 Prevents the Development of Experimental Graft-Versus-Host Disease

**Figure 1:**
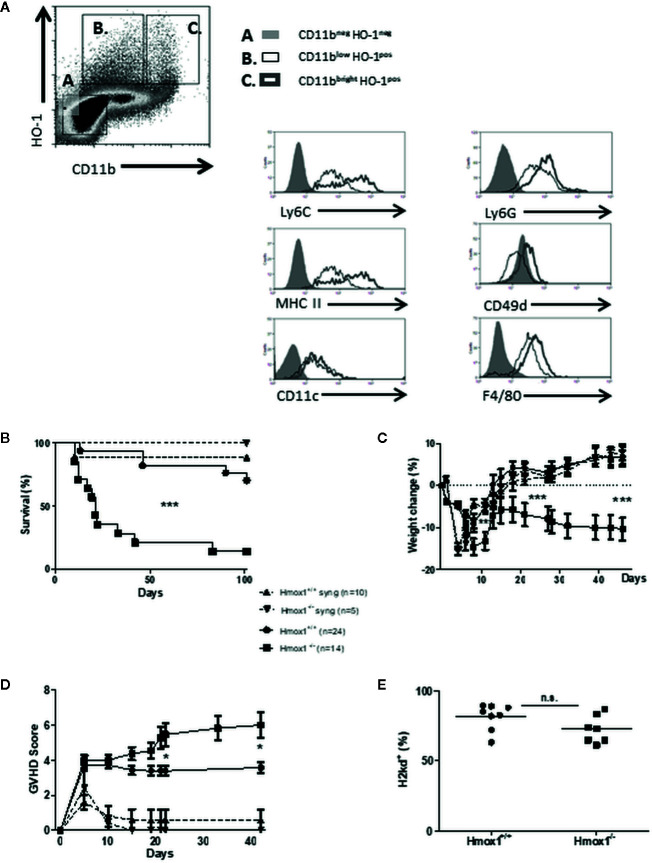
Donor-derived HO-1 regulates GVHD and survival. **(A)** BM cells from HSCT recipients were harvested on day 7 posttransplantation and characterized by FACS analysis. Two cell populations **(B, C)** expressing intracytoplasmic HO-1 were identified, based on CD11b, Ly6C, Ly6G, CD49d, F4/80, and MHC-II expression. Results are representative of five mice, seven days after transplantation. **(B–D)** GVHD characteristics were compared after syngeneic (BALB/c to BALB/c) and allogeneic (BALB/c to C57BL/6) BM transplantation. In lethally irradiated hosts, 5 × 10^6^ BM cells and 15 × 10^6^ spleen cells from either wild type (WT) or *Hmox1* ^−/−^ donors were injected. Survival **(B)**, % weight loss **(C)** and GVHD scores **(D)** were compared between groups. **(E)** Host reconstitutions by allogeneic (H2kd) BM and spleen cells are shown in peripheral blood monocytes (PBMCs). Three or four independent experiments involving 5–6 animals per group were pooled. n.s., no significant difference; *P <.05; **P <.01; ***P <.001.

We asked whether the expression of HO-1 was induced after experimental allogeneic HSCT and tested this hypothesis initially in BALB/c to C57BL/6 HSCT. Expression of HO-1 was induced in CD11b^+^Ly6C^high^CD49d^+^MHC-II^high^ monocytic MDSCs and in CD11b^+^Ly6C^low^MHC-II^low^ monocytes (**Figure 1A**). Both monocytic cell populations were F4/80^+^, CD11c^-^ and expressed intermediate levels of Ly6G (**Figure 1A**). This demonstrates that HSCT is associated with the induction of HO-1 in donor-derived hematopoietic cells.

Survival of C57BL/6 recipients transplanted with allogeneic BM and spleen cells from BALB/c mice was compromised when the donor cells lacked HO-1 expression (BALB/c *Hmox1* ^−/−^ donors), as compared to C57BL/6 recipients transplanted with donor cells expressing HO-1 (BALB/c *Hmox1^+/+^* donors) (**Figure 1B**). Survival in C57BL/6 recipients transplanted with allogeneic BALB/c *Hmox1* ^−/−^ BM and spleen cells was correlated with progressive body weight loss (**Figure 1C**) and clinical signs of GVHD (**Figure 1D**). This was not associated, however, with overall changes in hematopoietic chimerism (**Figure 1E**). Expression of HO-1 had no impact on the survival of syngeneic BALB/C recipients transplanted with BM and spleen cells from BALB/c *Hmox1* ^−/−^ or *Hmox1^+/+^* mice (**Figures 1B, D**). This demonstrates that donor-derived HO-1 expression is essential to prevent the development of allogeneic GVHD after HSCT.

**Figure 2:**
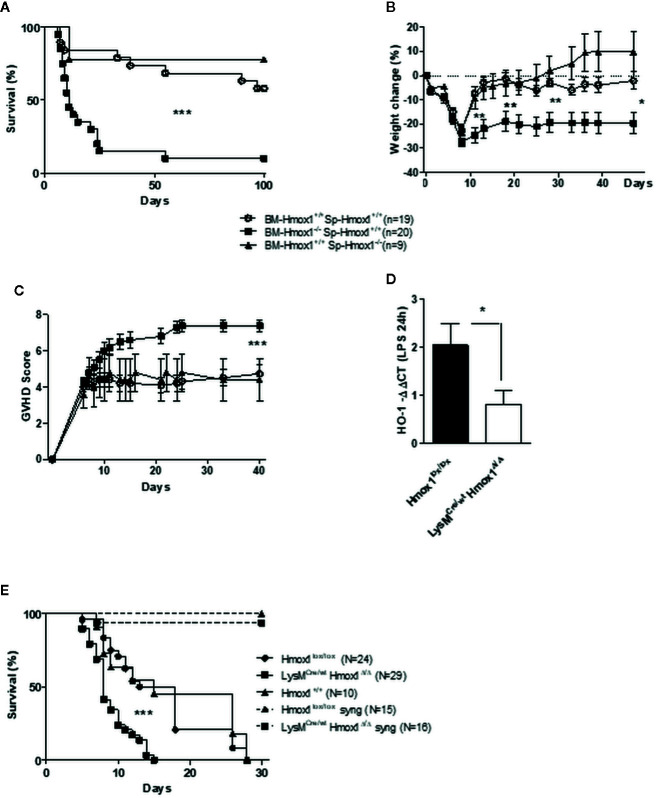
Myeloid HO-1 but not T cell-derived HO-1 regulates GVHD. Survival **(A)**, weight change **(B)** and GVHD score **(C)** were compared between C57BL/6 irradiated hosts transplanted with a mix of BM and T cells from wild type (*Hmox1* ^+/+^) BALB/c donors, with a mix of *Hmox1* ^−/−^ BM and *Hmox1* ^+/+^ BALB/c T cells, or with a mix of *Hmox1* ^+/+^ BM and *Hmox1* ^−/−^ BALB/c T cells, respectively. Results were pooled from three independent experiments including 6–7 mice per group. Donor chimerism (H-2Kd positive cells) was similar in both groups (data not shown). **(D–E)** A conditional HO-1 ablation in myeloid cells mimics the picture we observed with *Hmox1* ^−/−^ BM donors. The specific myeloid HO-1 deficiency was confirmed by comparing HO-1 transcription by lipopolysaccharide (LPS)-stimulated bone marrow-derived macrophages (BMDM) from control littermates *Hmox1^lox/lox^* or *LysM^Cre/wt^Hmox1^Δ/Δ ^*mice **(D)**. BMDM were generated as described in supplementary methods. Lethally-irradiated BALB/c mice were grafted with 2 × 10^6^ spleen cells from *Hmox1^lox/lox^* littermates and 2.5 × 10^6^ BM cells from either *Hmox1^lox/lox^* or *LysM^Cre/wt^Hmox1^Δ/Δ ^* donors. Three experiments each including 8–10 mice were pooled. **(E)** Transplantation of *Hmox1^lox/lox^* or *LysM^Cre/wt^Hmox1^Δ/Δ ^*BM cells into syngeneic C57BL/6 hosts did not trigger GVHD, in two separate experiments each with 7–8 mice. n.s. indicates no significant difference, *P <.05, **P <.01, ***P <.001.

We then assessed whether the protective effect exerted by HO-1 depends on the myeloid BM cells or the alloreactive spleen cells within the HSCT. Survival of C57BL/6 recipients was compromised only when the transplanted BM, but not spleen cells, were isolated from allogeneic BALB/c *Hmox1* ^−/−^ mice, as compared to control C57BL/6 recipients transplanted with allogeneic BM and/or spleen cells from BALB/c *Hmox1^+/+^* mice (**Figures 2A, C**). This suggests that expression of HO-1 by donor myeloid cells is required to suppress GVHD and all further experiments were performed using wild type *Hmox1^+/+^* spleen cells.

To prove unequivocally that expression of HO-1 by donor myeloid cells is required to suppress GVHD, we used C57BL/6 *LysM* ^Cre/wt^ *Hmox1^Δ/Δ^* mice in which the *Hmox*1 allele is deleted specifically in the myeloid compartment, as confirmed in LPS-stimulated BM-derived monocytic cells by qRT-PCR (**Figure 2D**). Incidence of lethality (**Figure 2E**) and GVHD severity (data not shown) were higher in BALB/c recipients transplanted with BM from C57BL/6 *LysM* ^Cre/wt^ *Hmox1^Δ/Δ^* mice, as compared to control BALB/c recipients transplanted with BM from wild type C57BL/6 *Hmox1^+/+^* or with C57BL/6 *Hmox1^flox/flox^* mice (**Figure 2E**). This was not the case for syngeneic C57BL/6 recipients transplanted with BM from C57BL/6 *LysM* ^Cre/wt^ *Hmox1^Δ/Δ^* mice, as compared to control C57BL/6 recipients receiving wild type *Hmox1^+/+^* or *Hmox1^flox/flox^* BM (**Figure 2E**).

### Donor Myeloid HO-1 Suppresses T Cell Alloreactivity After Experimental Hematopoietic Stem Cell Transplantation

**Figure 3:**
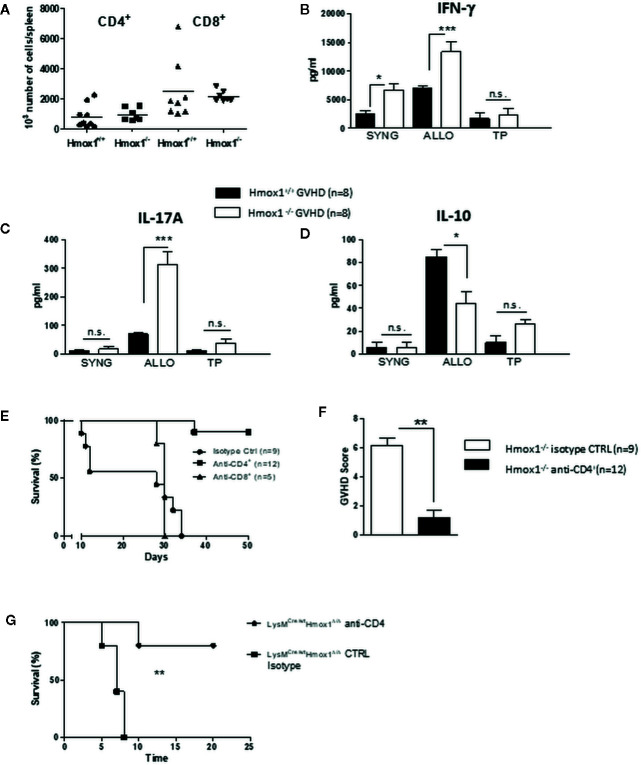
Donor-derived myeloid HO-1 controls T cell alloreactivity. **(A)** The number of donor-derived CD4+ and CD8+ T cells was quantified by flow cytometry at day 7 post-transplantation in C57BL/6 hosts grafted with *Hmox1* ^−/−^ BM and WT BALB/c T cells (*Hmox1* ^−/−^ GVHD) and compared with controls (WT GVHD). **(B–D)** Spleen cells from *Hmox1* ^−/−^ versus *Hmox1* ^+/+^ BM reconstituted hosts were cultured with donor type (SYNG: syngeneic BALB/c), host type (ALLO: allogeneic C57BL/6), or third party (TP) (CBA/Ca) irradiated stimulatory cells. IFN-γ, IL-17A, and IL-10 were measured in MLR supernatants. Two independent experiments involving 8 animals per group were pooled. **(E, F)** The effects of either CD4+ or CD8+ T cell depletion (see materials and methods) on host survival and GVHD score are shown in the BALB/c (*Hmox1* ^−/−^ versus *Hmox1* ^+/+^ BM) to C57BL/6 graft versus host combination. **(G)** The effect of CD4+ depletion on host survival in the BALB/c recipients transplanted with BM from C57BL/6 *LysM* ^Cre/wt^ *Hmox1^Δ/Δ^* mice. n.s., no significant difference; *P <.05; **P <.01; ***P <.001.

We then investigated the mechanism by which the expression of HO-1 in donor myeloid cells suppresses the development of GVHD. The number of donor-derived CD4+ and CD8+ T cells did not differ between recipients of either *Hmox1* ^−/−^ or *Hmox1* ^+/+^ BM donors (**Figure 3A**). To address whether HO-1 expression modulates T cell alloreactive responses underlying GVHD, we performed standard mixed lymphocyte reactions (MLR) using spleen cells harvested seven days after HSCT and syngeneic, allogeneic, or third-party stimulatory cells. Leukocytes isolated from C57BL/6 recipients transplanted with BM from BALB/c *Hmox1* ^−/−^ mice produced higher levels of IL-17 and IFN-γ while the production of IL-10 was reduced, as compared to leukocytes isolated from C57BL/6 recipients transplanted with BM from BALB/c *Hmox1^+/+^* mice (**Figures 3B, D**). Surprisingly, in the syngeneic cultures (left columns), IFN-γ production by leukocytes isolated from C57BL/6 recipients transplanted with BM from BALB/c *Hmox1* ^−/−^ mice was higher than that in leukocytes isolated from C57BL/6 recipients transplanted with BM from BALB/c *Hmox1^+/+^* mice (**Figure 3B**). It is possible that spleen cells that have been seeded in culture still contain allogeneic and alloreactive cells, allowing ex-vivo IFN-γ production independently of the MLR stimulatory condition. In line with this, the highest amounts of IFN-γ are produced by B6 hosts transplanted with Hmox1^−/−^ donors. This effect is observed with IFN-γ but not IL-17 or IL-10 due to the larger amounts of cytokine production.

Finally, the onset of acute GVHD aggravation in C57BL/6 recipients transplanted with BM from BALB/c *Hmox1* ^−/−^ mice was also reflected by the increase in IFN-γ serum levels, IL-17A or IFN-γ mRNA in the liver, serum aspartate aminotransferase (AST) levels, and oxidative stress-induced molecules (**Figures S1A, G**). Of note, this was not observed in the syngeneic conditions (data not shown).

The enhanced mortality in C57BL/6 hosts transplanted with BM from BALB/c *Hmox1* ^−/−^ mice was T cell-mediated since CD4+ T cell depletion dramatically prevented GVHD and mortality (**Figures 3E, F**). CD8+ T cell depletion also modulated GVHD, but to a lesser extent (**Figure 3E**). However, in the CD8+ T cell depletion experiments, the results should be interpreted cautiously due to the small number of animals included. A comparable effect of CD4 T cell depletion was observed in the BALB/c recipients transplanted with BM from C57BL/6 *LysM* ^Cre/wt^ *Hmox1^Δ/Δ^* mice (**Figure 3G**).

We then asked whether expression of HO-1 could modulate alloreactive cytotoxic CD8+ T cell (CTL) responses, a critical component in the pathogenesis of GVHD. CTL activity was compared *in vivo* in BALB/c recipients transplanted with BM from C57BL/6 *LysM* ^Cre/wt^ *Hmox1^Δ/Δ^* versus BM from wild type C57BL/6 *Hmox1^+/+^* or C57BL/6 *Hmox1^flox/flox^* mice. CTL activity was similar in all allogeneic BM transplant combinations, as assessed *in vivo* by using CFSE-labeled splenocytes corresponding to the transplanted BM genotype (**Figure S2**).

### Donor HMOX1 (GT)_n_ Polymorphism Is Associated With the Incidence and Severity of Acute Graft-Versus-Host Disease

In order to investigate the clinical relevance of the previous experimental data, we assessed the impact of donor and recipient HO-1 polymorphisms in the outcome of HSCT in patients.

A total of 120 and 160 HSCT procedures in two independent cohorts were investigated. The minimum follow-up was 1 year. **Tables 1** and **2** summarize patient and donor characteristics and treatments. In both cohorts, underlying diseases are listed according to their frequency. Types of transplantation have been divided into three categories: matched related donor (MRD), matched unrelated donor (MUD), and unmatched related donor (URD). Conditioning treatments were separated in two categories: myeloablative conditioning (MAC) and reduced intensity conditioning (RIC). The two cohorts differed in the following characteristics: (1) there was no transplantation from URD in the first cohort, versus 22% in the second one; (2) there was a higher proportion of RIC in the first cohort; (3) there was a lower rate of radiotherapy used in the first cohort; (4) GVHD prophylaxis with ATG was used in 25% of patients in the first cohort versus 76.2% in the second.

**Figure 4:**
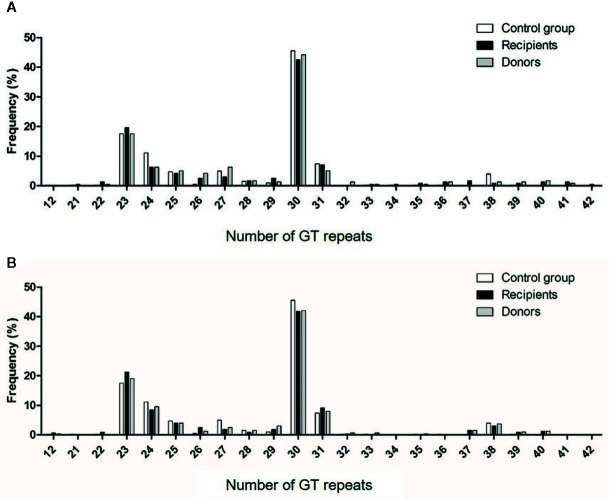
Distribution of the number of (GT)_n_ repeats in the microsatellite region of the HO-1 gene promoter. Numbers of (GT) repeats are shown in HSC donors (gray bars) and recipients (black bars) of cohorts 1 **(A)** and 2 **(B)** and in healthy volunteers (white bars).

The bimodal distribution of (GT)_n_ polymorphisms in the *HMOX1* promoter in donor and recipients from cohorts 1 and 2 and healthy controls (**Figures 4A, B**), ranged from 12 to 42 and peaked at 23 and 30 (GT)_n_, similar to that observed in previous studies (27–29, 35, 46, 47). The (GT)_n_ alleles were segregated into short (S), i.e. (GT)_n_ < 30, and long (L), i.e. (GT)_n_ > 30, and individuals were classified as L/L, L/S, or S/S genotypes, as described before (23). The distributions of S or L alleles and genotype frequencies were similar in recipients and donors, in both cohorts and in the control group (**Table 3**): Hardy-Weinberg equilibrium was met.

**Table 3:** Allele and genotype frequencies in the two cohorts and the control group.

	Cohort 1		Cohort 2		Control Group
	Recipient	Donor	Recipient	Donor	
**Allele Type**					
Short, n (%)	99/240 (41.3)	102/240 (42.5)	135/320 (42.1)	130/316 (41.1)	170/406 (41.9)
Long, n (%)	141/240 (58.7)	138/240 (57.5)	185/320 (57.9)	186/316 (58.9)	236/406 (58.1)
**Genotype**					
Short/Short, n (%)	18/120 (15.0)	23/120 (19.2)	34/160 (21.2)	31/158 (19.6)	32/203 (15.7)
Long/Short, n (%)	63/120 (52.5)	56/120 (46,7)	71/160 (44.3)	71/158 (44.9)	105/203 (51.7)
Long/Long, n (%)	39/120 (32.5)	41/120 (34.2)	55/160 (34.3)	56/158 (35.4)	66/203 (32.5)

Distribution of short (S) or long (L) alleles and genotype frequency in recipients, donors in cohorts 1 and 2 and in the control group.

*Allele and genotype are missing in two donors in cohort 2 due to technical reason.

As shown in **Table 4**, the proportion of L alleles in the recipient was not associated with GVHD severity, whereas a trend for a higher proportion of L alleles in the donor was observed in patients with grade III/IV acute GVHD (S vs L allele: 28.6% vs 71.4%, p=0.05 and 33.8 vs 66.2%, p=ns in cohorts 1 and 2, respectively). The L/L genotype in the donor was associated with a higher proportion of grade III/IV acute GVHD in both cohorts (S/S or S/L vs L/L: 46.4% vs 53.5%, p<0.05 and 47.0% vs 52.9% p<0.05 in cohorts 1 and 2, respectively), whereas an L/L genotype in the recipient did not influence GVHD severity (**Table 5**).

**Table 4:** Allele distribution according to GVHD severity.

Allele type	Cohort 1			Cohort 2		
	GVHD severity			GVHD severity		
**Recipient**	No GVHD	Grade 1–2	Grade 3–4	No GVHD	Grade 1–2	Grade 3–4
Short, n (%)	48 (41.4)	28 (46.7)	23 (35.9)	68 (43.0)	39 (52.7)	25 (34.7)
Long, n (%)	68 (58.6)	32 (53.3)	41 (64.1)	90 (57.0)	35 (47.3)	47 (65.3)
**Donor**						
Short, n (%)	54 (45.8)	32 (48.5)	16 (28.6)	70 (44.3)	36 (46.1)	23 (33.8)
Long, n (%)	64 (54.2)	34 (51.5)	40 (71.4)^#^	88 (55.7)	42 (53.8)	45 (66.2)

Number of short (S) and long (L) alleles and frequency in donors and recipients according to the grade of GVHD. L for (GT)_n_ longer than 30 repeats and S for (GT)_n_ shorter than 30 repeats. ^#^p = 0.05 compared to moderate or no GVHD. GVHD, graft-versus-host disease.

*Clinical data for GVHD severity are incomplete for eight patients in cohort 2.

**Table 5:** Genotype distribution according to GVHD severity.

Genotype	Cohort 1			Cohort 2		
	GVHD severity			GVHD severity		
**Recipient**	No GVHD	Grade 1–2	Grade 3–4	No GVHD	Grade 1–2	Grade 3–4
S/S or S/L, n (%)	40 (69)	22 (73.3)	19 (59.4)	54 (68.4)	27 (73)	18 (50)
L/L, n (%)	18 (31)	8 (26.7)	13 (40.6)	25 (31.6)	10 (27)	18 (50)
**Donor**						
S/S or S/L, n (%)	42 (71.1)	24 (72.7)	13 (46.4)	55 (69.6)	28 (71.7)	16 (47.0)
L/L, n (%)	17 (28.8)	9 (27.7)	15 (53.5)*	24 (30.3)	11 (28.2)	18 (52.9)*

Proportion of L/L genotype in hematopoietic stem cell in donors and recipients according to the grade of GVHD in cohorts 1 and 2; *p <0.05 compared to moderate or no GVHD. GVHD, graft-versus-host disease. For the L/L genotype in the recipient, p value is not statistically significant: p=0.48 in cohort 1, p=0.08 in cohort 2.

*Clinical data for GVHD severity are incomplete for eight patients in cohort 2.

The association between GVHD and risk factors was analyzed by univariate and multivariate analyses, considering L/L donor genotype, age, cytomegalovirus (CMV) status, gender (donor and recipient), type of transplantation, intensity of conditioning, and use of radiotherapy (**Tables 6** and **7**). Subdistribution hazard ratio (SHR) for grade III/IV acute GVHD was significantly higher in cases involving an L/L genotype in the donor (SHR: 2.3; CI: 1.2–4.4, p=0.01 and SHR: 2.0; CI: 1.1–3.6, p=0.02, in cohorts 1 and 2, respectively) and of an L/L genotype in the recipient only in cohort 2 (SHR: 2.0; CI: 1.1–3.6; p=0.02). Other acknowledged risk factors for developing GVHD (48) remained significant after multivariate analysis, such as radiotherapy or the type of transplantation (**Table 7**). Radiotherapy was a risk factor for GVHD in cohort 2 but not in cohort 1, in which radiotherapy was much less frequently used. Graft origin (MUD) was identified as a risk factor for GVHD in cohort 1 but not in cohort 2, probably related to the systematic use of ATG (**Tables 6** and **7**). Regarding ATG use, multivariate analysis was not performed because univariate analysis did not find statistical correlation when performed in the cohort with the most abundant use of ATG (data not shown). Importantly, multivariate analysis revealed that the donor’s L/L genotype remained a significant risk factor for GVHD, independently of radiotherapy and of the graft source (aSHR: 2.6; CI: 1.3–4.9, p=0.005 and aSHR: 1.9; CI: 1.0–3.5, p=0.04 for cohorts 1 and 2, respectively). In summary, HSCT from a donor harboring the L/L genotype increases the risk for developing severe acute GVHD. This corroborates our experimental data in mice, strongly suggesting that the relative level of HO-1 expression in donor BM plays a critical role in the onset of GVHD.

**Table 6:** Univariate analysis of GVHD risk factors.

	Cohort 1		Cohort 2	
	SHR (95%)	p-value	SHR (95%)	p-value
**Sex**				
Male	1.05 (0.55–1.98)	0.88	1.09 (0.57–2.06)	0.80
Female	1.0		1.0	
**Recipient Age** < 40	1.63 (0.88–3.02)	0.12	1.32 (0.74–2.38)	0.35
≥ 40	1.0		1.0	
**Donor** age < 40	1.15 (0.60–2.20)	0.67	1.26 (0.63–2.53)	0.51
≥ 40	1.0		1.0	
**Sex mismatch**				
Donor fem/male	0.78 (0.35–1.77)	0.56	0.79 (0.36–1.74)	0.56
Others	1.0		1.0	
**CMV mismatch**				
D-/R+	1.04 (0.42–2.58)	0.94	1.32 (0.66–2.63)	0.44
Others	1.0		1.0	
**Disease stage**				
CR1 or chr. ph. CML	1.0	0.27	1.0	0.90
>CR1 or ac. ph. CML	1.43 (0.76–2.67)		0.96 (0.53–1.74)	
**Graft**				
MRD	1.0	**0.004**	1.0	0.99
MUD	2.94 (1.42–6.12)		0.98 (0.46–2.07)	
URD	NA		1.04 (0.50–2.15)	
**Conditioning**				
RIC	1.0	0.77	1.0	0.26
MAC	0.91 (0.48–1.71)		0.70 (0.37–1.31)	
**Radiotherapy**				
No	1.0	0.36	1.0	**0.01**
Yes	1.42 (0.67–3.00)		2.20 (1.88–4.06)	
**Donor genotype**				
SS and SL	1.0	**0.015**	1.0	**0.02**
LL	2.28 (1.18–4.41)		2.00 (1.10–3.65)	
**Recipient genotype**				
SS and SL	1.0	0.30	1.0	**0.02**
LL	1.39 (0.75–2.56)		2.00 (1.12–3.56)	

SubHazard Ratio (SHR) of association of severe GVHD (grade III-IV) according to risk factors, NA: not applicable; CMV, cytomegalovirus; GVHD, graft-versus-host disease; MRD, matched related donor; MUD, matched unrelated donor; URD, unmatched related donor; MAC, myeloablative conditioning; RIC, reduced intensity conditioning.

**Table 7:** Multivariate analysis of GVHD risk factors.

	Cohort 1		Cohort 2	
	(cases = 28/n = 120)		(cases = 33/n = 151)	
	aSHR (95%)	p-value	aSHR (95%)	p-value
**Graft**				
MRD	1.0	**0.006**	1.0	0.81
MUD	2.72 (1.33–5.58)		0.81 (0.35–1.88)	
URD	NA		0.59 (0.37–1.76)	
**Radiotherapy**				
Yes	1.0	0.18	1.0	**0.02**
No	1.82 (0.76–4.38)		2.15 (1.11–4.18)	
**Donor genotype**				
SS and SL	1	**0.005**	1.0	**0.04**
LL	2.57 (1.34–4.94)		1.90 (1.04–3.5)	

Adjusted SubHazard Ratio (aSHR) of the association of severe GVHD (grade III-IV) according to risk factors. GVHD, graft-versus-host disease; MRD, matched related donor; MUD, matched unrelated donor; URD, unmatched related donor.

*Clinical and/or genetic data are incomplete for nine patients in cohort 2.

## Discussion

In this study, we provide evidence that donor-derived myeloid HO-1 prevents the onset of acute GVHD after HSCT. Importantly, the protective effect against GVHD associated with the expression of HO-1 was determined *via* a genetic loss-of-function approach, as compared to previous studies in which non-specific pharmacological induction of HO-1 was shown to be associated with suppression of GHVD (18–20). It should be noted, however, that these studies have not provided irrefutable evidence that the beneficial effects associated with these pharmacological approaches are indeed driven by the upregulation of HO-1 expression (18–20).

HO-1 can be stress-induced in almost all cell types, including epithelial or endothelial cells (49). In our mouse models, host animals are always in a wild type background and can upregulate HO-1 normally in response to many stress types, including GVHD. We have measured plasma HO-1 levels by ELISA and detected large amounts of plasma HO-1, whether the BMT donor was HO-1 sufficient or deficient (data not shown). Nevertheless, that HO-1 production did not afford any protection against GVHD, in contrast to the HO-1 from the BM donor.

Another study that enhanced expression of HO-1 in mesenchymal stem cells (MSC) by lentiviral gene transfer in a mouse GVHD model, showed a significant diminution of clinical and pathological GVHD scores in mice, but not on survival (50). As such, the current study is the first demonstration that expression of HO-1 in myeloid cells suppresses GVHD after HSCT in mice, and likely in humans as well, as suggested by the association between a (GT)_n_ L/L genotype associated with low HO-1 expression and GVHD severity.

Another study in HSCT patients that investigated the association between *HMOX1* (GT)_n_ promoter polymorphisms and transplantation-related mortality reported results that are in apparent contrast to ours (51). Gerbitz et al. (2008) investigated 92 patient-donor pairs that were classified as either high or low HO-1 expressors based on the presence or the absence of a single long allele (≥ 30 GT repeats). This “dominant” approach differs from the “recessive” approach we adopted (to be considered a low expressor requires the L/L genotype). In their study, Gerbitz et al. (2008) showed that HSCT from matched unrelated donors defined as high HO-1 expressors negatively influenced overall survival and transplantation-related mortality. Regarding the risk of acute GVHD, there was only a trend but no statistical difference, with a higher rate of grade III/IV acute GVHD when the unrelated donor was a low HO-1 expressor. This apparent discrepancy may result from cohort characteristics (only matched donor/recipient pairs), genotype definition (L allele dominance assumed), and selected outcomes. Indeed, transplantation-related mortality and overall survival are composite outcomes and the higher mortality observed by Gerbitz et al. (2008) might be due to increased susceptibility to infections and disease relapse rather than GVHD (23, 27–29, 52). Another study observed a higher incidence of grade III/IV acute GVHD in the donor L/L genotype group in the non MAC regimens cohort but not in the MAC regimens cohort (53). In the MAC cohort, they also found a significantly higher relapse incidence in the donor S/S genotype group, but no difference in overall survival. In this study, they did not observe the same effect of *HMOX1* (GT)_n_ promoter polymorphisms in their two cohorts and this may be due to the small size of the cohorts. A recent study that investigated the influence of the single nucleotide polymorphism (SNP) rs2071746 (-413A>T) in the HO-1 promoter on HSCT outcomes in 593 patients with hematological malignancies showed that the donor A/A or A/T genotype was associated with better 5-year overall survival and disease-free survival compared to the donor T/T genotype. The A allele has been associated with higher *HO-1* expression levels than the T allele. The donor HO-1 genotype showed no effects on GVHD (54). This apparent discrepancy might be explained by the use of different genotyping methodologies (SNP versus (GT)_n_ polymorphism) and we did not assess the incidence of overall survival, non-relapse mortality, and GVHD relapse-free survival.

There are potentially multiple overlapping mechanisms by which myeloid HO-1 can prevent the onset of severe GVHD (55). In line with previous observations (17), we demonstrate that expression of HO-1 by myeloid cells regulates alloreactive T cell responses in a manner that suppresses the onset of GVHD, suggesting, therefore, that pharmacological induction of HO-1 in this cell compartment may be used as a therapeutic approach in the treatment of GVHD. This finding is also in keeping with previous observations indicating that HO-1 activity is essential for and promotes tolerance to transplanted allogeneic organs (56). In accordance with this notion, we found that when expressed by myeloid cells, HO-1 suppresses alloreactive Th1 and Th17 cell responses involved in the onset of GVHD. Notably, when expressed in a non-myeloid compartment, HO-1 was unable to control these alloreactive responses.

The control of anti-host alloreactive T cells *via* HO-1 is in agreement with our previous observation that MDSCs can suppress alloreactive T cell responses and skin allograft rejection through an HO-1-dependent mechanism. This suggests that physiologic processes by which MDSCs control adaptive immunity can be regulated *via* HO-1-dependent mechanisms (17). The exact mechanism by which HO-1 exerts these effects is not clear and is likely to be multifactorial.

One possibility could be that HO-1 generates one or several metabolic products that act in MDSCs and/or directly on alloreactive T cells to suppress their activation and proliferation. In keeping with this notion, pharmacologic blockade of HO-1 activity is associated with the accumulation of reactive oxygen species (ROS) levels in dendritic cells (DCs) and, through a p38 mitogen-activated protein kinase (p38 MAPK)-dependent pathway, induces DC maturation and CD8+ T cell activation (57, 58).

Another possibility could be that under stress conditions, such as those associated with transplantation, HO-1 becomes the rate-limiting factor in the catabolism of heme, generating equimolar amounts of biliverdin, carbon monoxide (CO), and labile iron (59). Heme can be released from hemoproteins, under stress conditions, such as to generate labile heme (60). Labile heme is recognized by pattern recognition receptors, including toll-like receptor 4 and NACHT, LRR, and Pyrin Domain Containing 3 (NLRP3) (60) acting as a bona fide alarmin that activates monocyte/macrophages. Whether these effects of labile heme promote T cell alloreactivity and, hence, the rejection mediated by transplanted cells, tissues, or organs has not been established. What is clear, however, is that heme catabolism by HO-1 counters the pro-oxidant effects of labile heme in a manner that inhibits its pro-inflammatory and cytotoxic effects. Moreover, heme catabolism by HO-1 generates CO, which limits the production of labile heme while regulating monocyte/macrophage activation in a manner that inhibits the production of cytotoxic cytokines, e.g. TNF, while promoting that of immunoregulatory cytokines, e.g. IL-10 (58, 59). Macrophages are considered principal regulators of immune homeostasis and have been categorized into two subtypes: M1, which are proinflammatory *via* a Th1 response, and M2, which harbor an anti-inflammatory phenotype *via* a Th2 response (61, 62). Upregulation of HO-1 promotes M2 polarization (63–65). In line with this, Zhang et al. (2018) recently reported that M2 polarization through myeloid HO-1 partially prevents ischemia-reperfusion injury (66). In our GVHD model, it is not excluded that donor myeloid HO-1 controls T cell alloreactivity through M2 polarization. Another group has shown that monocytes co-cultured with expanded T_REGs_ downregulate costimulatory and MHC-class II molecules and upregulate M2 markers, including HO-1 and IL-10 (67).

We very recently observed in a mouse tumor model that the deletion of HO-1 in the myeloid compartment enhances the beneficial effects of a therapeutic antitumor vaccine by boosting CD8 T cell proliferation and cytotoxicity (68). This underlines the possible modulation of adaptive immune response through myeloid HO-1. In addition, myeloid HO-1 deficiency induced a modulation of tumor-associated macrophage transcriptional and epigenetic programs. Comparable mechanisms might be involved in our GVHD model.

An intriguing possibility is that MHC class I-restricted presentation of HO-1-derived peptides exerts immunoregulatory effects on alloreactive T cells, much like that demonstrated in cancer patients, where HLA-A2–restricted CD8+ T_REGs_ specific for HO-1-derived peptides suppress tumor specific T cell responses (69). Nevertheless, our data suggests that CD8+ T cell depletion does not protect against GVHD in mice receiving BM allogeneic transplants in which the expression of HO-1 is deleted in myeloid cells, which does not fully support that an immunoregulatory mechanism based on CD8+ T_REG_ to be operational here.

It is also possible that the protective effect of HO-1 could act *via* additional mechanisms such as the CO generated by heme catabolism which promotes myeloid cell expansion and differentiation (70). In addition, CO exerts potent cytoprotective (71) and anti-inflammatory effects (72, 73) that should restrain the severity of GVHD as well.

Expression of HO-1 in myeloid cells may also play a critical role in restoring iron metabolism after transplantation. In support of this notion, Kovtunovych et al. reported that subablative BMT cured *Hmox1* ^−/−^ mice by repopulating tissue macrophages in a syngeneic context, restoring iron metabolism parameters, and by normalizing oxidative stress-induced molecules in the liver and the kidney (74). Nevertheless, in our experiments, the lack of HO-1 in syngeneic BMT only marginally affected outcomes, which underlines the synergy of alloantigens in the pathogenicity of myeloid-restricted HO-1 deficiency. Based on the results presented here and those of others, it might be reasonable to consider HO-1 induction, either to prevent GVHD or as adjuvant therapy to treat GVHD. Indeed, a broad range of HO-1 inducers that have been recently described (75, 76) might be used in the clinic.

We conclude that donor-derived myeloid HO-1 is a key regulator of T cell alloreactivity and acute GVHD severity and constitutes a potential therapeutic target for HSCT patients. Large-scale prospective studies in HSCT patients are necessary to validate the HO-1 L/L genotype as an independent risk factor to developing severe acute GVHD. Additional mechanistic approaches should allow a better understanding of how HO-1 regulates GVHD and T cell functions. If the role of HO-1 is confirmed, the HO-1 (GT)_n_ polymorphism of the donor might be considered as a biomarker before BMT in a personalized medicine strategy combined or not with therapeutic use of HO-1 inducers.

## Data Availability Statement

The datasets presented in this study can be found in online repositories. The names of the repository/repositories and accession number(s) can be found in the article/**Supplementary Material**.

## Ethics Statement

The studies involving human participants were reviewed and approved by Erasme Hospital and the Jules Bordet Institute (numbers P2011/255 and 2012/193, respectively). The Henri Mondor University Hospital (Créteil, France) has been accredited according to the international JACIE program since 2005. All patients and donors signed informed consent for (1) registration into the Promise database, and (2) cryopreservation of their biological material for research purposes. Written informed consent for participation was not required for this study in accordance with the national legislation and the institutional requirements. The animal study was reviewed and approved by local committee for animal welfare (agreement # LA2500519).

## Author Contributions

CS, VW designed and performed the research, analyzed the data, and wrote the paper. BV, J-MH, and VF helped to perform the research. PL, LC, CR, FB, and DB provided the materials and performed the research. JR performed the statistical analysis and wrote the paper. MA critically reviewed the manuscript and analyzed the genetic data. MS provided the material and critically reviewed and wrote the manuscript. SM and AL designed the research, analyzed the data, and wrote the manuscript. All authors contributed to the article and approved the submitted version.

## Funding

CS was also funded by the Société Francophone de Transplantation (France), the Alice and David Van Buuren Foundation, and the Fonds Carine Vyghen pour le don d’organes. MS is supported by Fundação Calouste Gulbenkian and by competitive grants from the Fundação para a Ciência e Tecnologia (PTDC/SAU TOX/116627/2010, HMSP-ICT/0022/2010) and by the European Community 7th Framework Grant ERC-2011-AdG. 294709.

## Conflict of Interest

The authors declare that the research was conducted in the absence of any commercial or financial relationships that could be construed as a potential conflict of interest.
