# Supplementary material for: Donor-Derived Myeloid Heme Oxygenase-1 Controls the Development of Graft-Versus-Host Disease
Source: Front Immunol. 2021 Jan 18;11:579151. doi: 10.3389/fimmu.2020.579151 (PMC7849683; doi:10.3389/fimmu.2020.579151)
Supplement: Supplementary file 1 [file DataSheet_1.docx]

**SUPPLEMENTARY DATA**

**Materials and methods**

**Myeloablative regimens**

Regimens were as follows: In cohort 1, standard myeloablative conditioning regimen combined either 12 Gray (Gy) total body irradiation (TBI), fractionated over three days, with a total dose of cyclophosphamide of 120 mg/kg administered over two days, or a total dose of intravenous busulfan of 12.8 mg/kg (16 mg/kg, if oral) administered over four days with a total dose of cyclophosphamide of 120 mg/kg administered over two days. The standard regimen was modified in eight patients because of underlying disease or poor medical condition. Standard reduced-intensity conditioning (RIC) combined either a total dose of 90 mg/m² of [fludarabine](http://www.uptodate.com/contents/fludarabine-drug-information?source=see_link) administered over three days with low dose TBI (2 or 4 Gy), or [fludarabine](http://www.uptodate.com/contents/fludarabine-drug-information?source=see_link) 150 mg/m^2^ (total dose) administered over four days with oral [busulfan](http://www.uptodate.com/contents/busulfan-drug-information?source=see_link) (8 to 10 mg/kg) administered over three days, or fludarabine 120 mg/m^2^ (total dose) administered over four days and cyclophosphamide 1000 mg/m^2^ administered over 3 days. Immunosuppression for GVHD prophylaxis combined cyclosporine with methotrexate for myeloablative transplants, or cyclosporine with mycophenolate mofetil for RIC transplants. In vivo, T cell depletion, obtained with antithymocyte globulin (ATG), was added to the conditioning for RIC transplants and for two others undergoing myeloablative conditioning for hemoglobinopathy. In the second cohort, 160 patients underwent allogeneic stem cell transplantation at the Jules Bordet Institute in Brussels (Belgium) between January 2001 and December 2011. Grafts were from HLA-matched related donors (MRD), or HLA-matched unrelated donors (MUD), or haploidentical related donors (5/10 HLA-unmatched related donor (URD). The standard myeloablative conditioning regimen combined 12 Gy TBI fractionated over four days with cyclophosphamide 120 mg/kg (total dose) administered over two days or intravenous busulfan 12.8 mg/kg total dose (16 mg/kg, if oral) administered over four days and cyclophosphamide 120 mg/kg total dose administered over two days. For URD, standard conditioning combined fludarabine 200 mg/m^2^ total dose administered over five days, melphalan 120 mg/m^2^ total dose administered over two days, and 10 Gy fractionated TBI. Standard RIC combined fludarabine 120 mg/m^2^ total dose administered over four days and either cyclophosphamide 1000 mg/m^2^ administered over 3 days or a 2Gy single dose TBI. Immunosuppression, for GVHD prophylaxis, included standard doses of cyclosporine and methotrexate for MRD and MUD myeloablative transplants, or cyclosporine and mycophenolate-mofetil for RIC transplants. For haploidentical transplants, ex vivo T cell depletion was obtained by a donor CD34+ stem cell positive selection performed on Clinimacs (Miltenyi Biotec). The CD3 target number was ≤ 5x10^4^ cell/kg. Posttransplant, the patients did not receive immunosuppressive drugs. In vivo, T cell depletion, obtained with ATG (5 mg/kg) from day -6 to day -1, was included in the myeloablative conditioning in patients transplanted with MUD or URD. All patients going through an RIC transplant received ATG (10 mg/kg) on day -4 and day -3 before transplant.

**Table S1**. **Forward and reverse primer sequences and probes**

| **Gene** | **Forward** | **Reverse** | **Probe** |
| --- | --- | --- | --- |
| β-actin | CCGAAGCGGACTACTATGCTA | TTTCTCATAGATGGCGTTGTTG | ATCGGTGGCTCCATCCTGGC |
| IFN-γ | GGATGCATTCATGAGTATTGC | GCTTCCTGAGGCTGGATTC | TTTGAGGTCAACAACCCACAGGTCCA |
| IL-17A | GCTCCAGAAGGCCCTCAG | CTTTCCCTCCGCATTGACA | ACCTCAACCGTTCCACGTCACCCTG |
| HO-1 | GCCGAGAATGCTGAGTTCAT | AGGAAGCCATCACCAGCTTA | AGAACTTTCAGAAGGGTCAGGTGTCCA |
| Gstm1 | GAAGCCAGAGTTCTTGAAGACCATCC | AAGCAAGGAAATCCACATAGGTGACC | TCTACTCTGAGTTCCTGGGCAAGAGGC |
| Gsta2 | CTGACTGAAATGATTGGGCAATTGG | CAAAGGCAGGCAAGTAACGG | CCAGACCAAAGAGAAGCCAAGACTGCC |
| Gclc | AGCCTCCTCCTCCAAACTCAGA | GTTCTCGTCAACCTTGGACAGCG | AGAGTTCCGACCAATGGAGGTGCAGT |

**Figure S1. Donor-derived myeloid HO-1 controls liver damage.** (A) IFN-γ amounts in sera of hosts grafted with WT or *Hmox1*^-/-^ allogeneic BM cells. IFN-γ was undetectable in non-transplanted mice (data not shown). Results from three experiments were pooled. (B-C) Liver IL-17A and IFN-γ mRNA levels, results from two experiments were pooled. (D-G) Serum aspartate aminotransferase (AST) and liver Gstm1, Gsta2, and Gclc mRNA compared between groups. Mice from three experiments were pooled. n.s.: no significant difference; *: *P* <.05; **: *P* <.01; ***: *P* <.001.

**Figure S2. Impact of HO-1 on anti-host cytotoxicity.** C57BL/6 mice were transplanted with either *Hmox1^+/+^* (n = 5) or *Hmox1^-/-^* (n = 4) BALB/c bone marrow cells. Five days later, equivalent amounts of allogeneic (C57BL/6) and syngeneic (BALB/c) fluorescent targets were injected simultaneously in each recipient. Spleen cells were collected 2 hours later for flow-cytometry analysis. The results are expressed as percentages of cytotoxicity (mean ± SEM).n.s.: no significant difference.

**Figure S1.** **Donor-derived myeloid HO-1 controls liver damage**


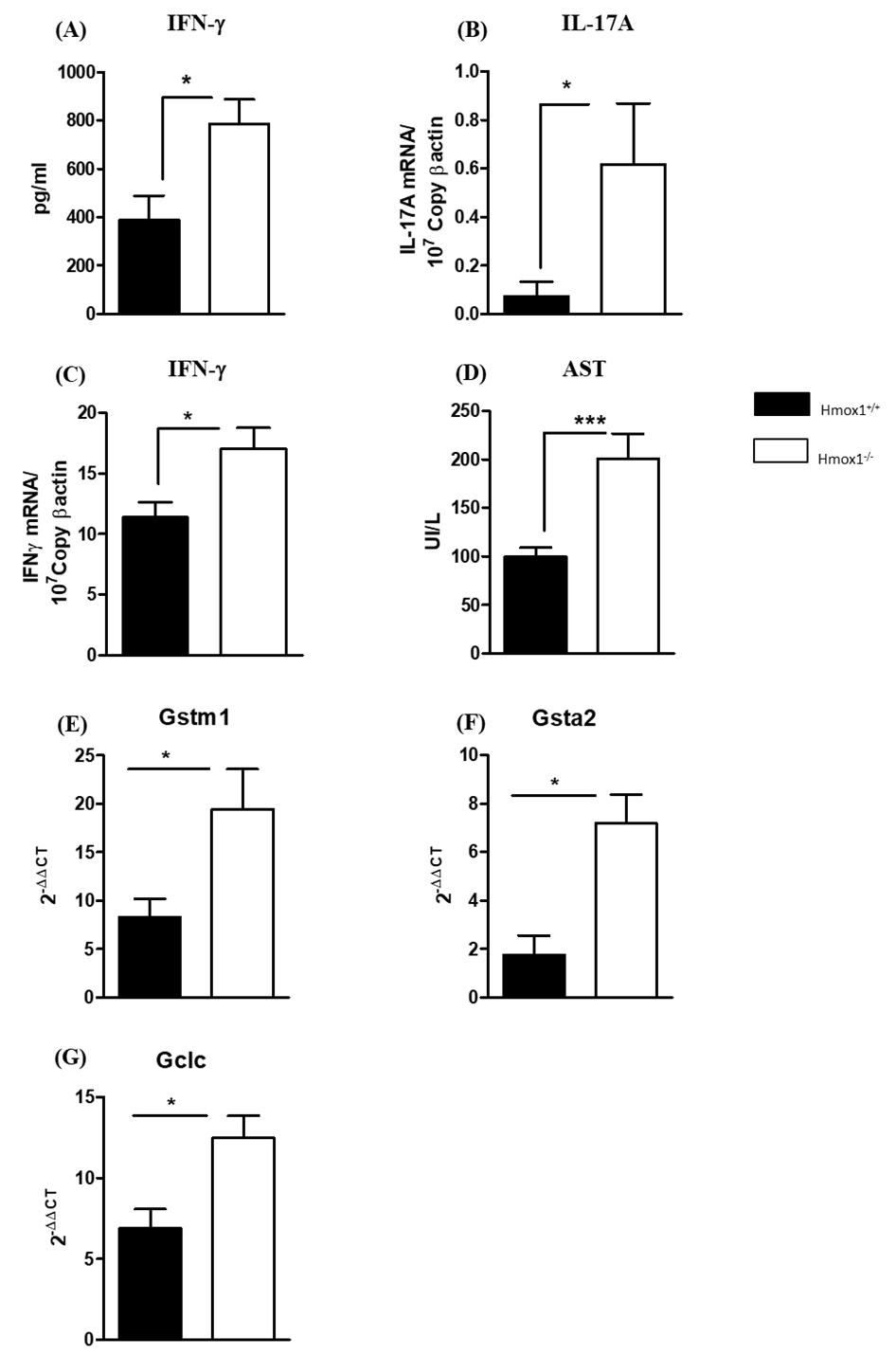


**Fig. S2.** **Impact of HO-1 on anti-host cytotoxicity**
